# Community willingness to participate in prehospital injury care: A cross-sectional survey of injury-prone areas along the national 3 highway in Cameroon

**DOI:** 10.1371/journal.pone.0332179

**Published:** 2025-09-11

**Authors:** Elvis Asangbeng Tanue, Casey Chun, Alan Hubbard, OConnor Kathleen, Odette D. Kibu, Serge Ngekeng, Nicholas Tendongfor, Nahyeni Bassah, Sandra I. McCoy, Rasheedat Oke, Isaac Obeng-Gyasi, Georges Nguefack-Tsague, Dickson S. Nsagha, Catherine Juillard, Alain Chichom-Mefire, S. Ariane Christie

**Affiliations:** 1 Sustainable Trauma, Research, Education and Mentorship (STREaM) project, Faculty of Health sciences, University of Buea, Buea, Cameroon; 2 Department of Public Health and Hygiene, Faculty of Health Sciences, University of Buea, Buea, Cameroon; 3 Program for the Advancement of Surgical Equity, Department of Surgery, University of California Los Angeles, Los Angeles, California, United States of America; 4 Division of Epidemiology, School of Public Health, University of California Berkeley, California, United States of America; 5 Department of Public Health, Faculty of Medicine and Biomedical Sciences, University of Yaoundé I, Yaoundé, Cameroon; 6 Data Science Center for the Study of Surgery, Injury, and Equity in Africa (D-SINE Africa), University of Buea, Buea, Cameroon; KPC Medical College and Hospital, INDIA

## Abstract

**Background:**

Road traffic injuries (RTIs) are a growing public health problem requiring urgent attention in Cameroon where emergency medical services (EMS) are underdeveloped. In other countries, training laypersons to provide prehospital care has been shown to improve injury outcomes, but requires buy-in from the persons being trained to provide care. To inform development of a lay first responder (LFR) program in Cameroon, this study aimed to assess the willingness of community members and associated factors to provide prehospital care for RTIs along the N3 highway, a road linking Cameroon’s two largest cities, known to have high incidence of RTIs.

**Methods:**

We conducted a cross-sectional survey of community members living along the N3 highway, between June 18^th^ and August 16^th^, 2024. Health district officials and community leaders identified N3 communities across 11 health districts with high rates of RTI. Purposeful sampling was performed in each community to assess exposure to injury and willingness to participate in prehospital care. Trained research assistants verbally administered a structured questionnaire to each consenting household representative; data collected included socio-demographic characteristics, injury exposure, first aid knowledge and attitudes, and willingness to provide prehospital care to victims of RTIs. Associations between demographic factors and willingness to provide prehospital care were assessed using multivariable logistic regression. Data were analyzed using IBM-SPSS version 26.0 and statistical significance was set at p < 0.05.

**Results:**

A total of 449 adult community members were surveyed. Most [268 (59.7%)) respondents were male with a median age of 33 years (interquartile range: 26–40). The majority, 333 (74.6%) community members were willing to provide care to injured victims. However, a third [167 (37.2%)] had adequate knowledge (scored ≥ 80%) of first aid and only 23 (5%) had been trained in first aid. Factors independently associated with willingness to provide prehospital care included having adequate first aid knowledge (adjusted odd ratio (aOR) = 1.69, 95% confidence interval (CI): 1.01–2.81, p = 0.046), primary education (aOR=4.20, 95% CI: 1.19–4.81, p = 0.026) and secondary education (aOR=4.70, 95% CI: 1.34–16.53, p = 0.016) compared to respondents with no formal education, prior witness of RTI (aOR=1.68, 95% CI: 1.055–2.68, p = 0.028), being aged between 30 and 40 years (aOR=1.82, 95% CI: 1.06–3.14, p = 0.031) and community members being able to call dedicated phone numbers to report RTIs (aOR=3.11, 95% CI: 1.28–7.54, p = 0.012).

**Conclusion:**

Most community members living in injury exposed-communities reported willingness to participate in prehospital care. However, first-aid knowledge is currently lacking in these communities. LFR training is needed in these communities to enable willing community members to contribute to prehospital efforts for RTIs along this road network.

## Introduction

Injuries cause over 4.4 million deaths annually worldwide, accounting for nearly 8% of all fatalities, and are the leading cause of death for individuals under 40, disproportionately affecting low- and middle-income countries (LMICs) [1,2]. An estimated 45% of injury deaths could potentially be prevented with mature pre-hospital care systems but many LMICs, including Cameroon, lack formal emergency medical services (EMS) [3–5]. In low-resource settings where access to professional prehospital care may be scarce, the World Health Organization (WHO) advocates for lay first responder (LFR) programs in the development of an EMS system [6,7]. LFR programs are designed to train community members in providing first aid, enabling them to offer immediate life-saving interventions before the patient reaches formal medical care [8]. However, these programs critically rely on cultural fit and participant buy-in to be effective; without community investment, the effectiveness and sustainability of LFR programs is greatly limited.

LFR programs in sub-Saharan Africa have most commonly concentrated on training cohorts with high injury exposure including commercial drivers [9–11]. However, stakeholder interviews have identified significant barriers to targeting commercial drivers as a principle LFR training target in Cameroon, including the financial losses that drivers would incur from participation and strained relations with the healthcare community [12]. To date, there has been limited exploration of LFR acceptability among individual who are not commercial drivers or police officers. Additional data are needed to assess the contextual appropriateness of training other potential cohorts.

The Yaoundé-Douala-Idenau National Road (N3) is the principal transportation corridor that connects Yaoundé (the political capital) and Douala (the economic capital) to the tourist hubs of Limbe in the south-western part of the country. The N3 is one of the most dangerous roads on the planet [13] with a road traffic injury (RTI) fatality rate that is 35 times higher than highways in Europe and the United States [14]. Communities along the N3 have extremely high exposure to injuries and, due to proximity, individuals from these communities are often the first to arrive at and respond to road traffic crashes, making them a promising potential cohort for LFR training. However, no prior data exists addressing LFR acceptability among the residents of these N3-adjacent communities. As part of a larger effort towards developing a scalable LFR prehospital care program for Cameroon, our study aimed to determine the willingness of N3 community residents in engaging with a LFR program and to investigate factors associated with willingness to provide prehospital care interventions in response to RTIs.

## Methods

### Study design

We conducted a cross-sectional survey involving adult residents sampled from injury-prone communities along the N3. The survey took place from the 18^th^ of June to the 16^th^ of August 2024.

### Study area and target population

**Fig 1:**
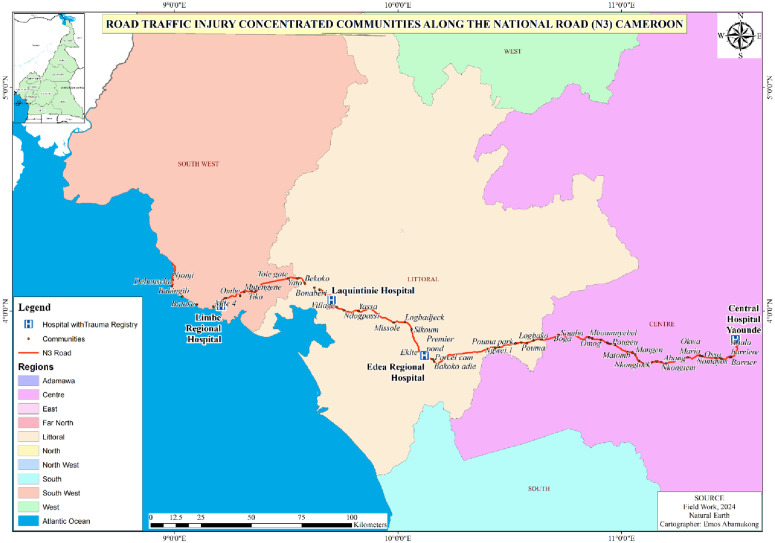
Road traffic injury concentrated communities along N3 in Cameroon.

The N3 has the highest incidence of road traffic injuries in Cameroon. Between January 1 and July 1, 2024, there were 3,413 road traffic accidents on the Yaoundé-Douala highway, resulting in 256 deaths and 254 injuries. These road traffic crashes also led to 2,570 cases of property damage and impacted 635 individuals across 208 fatal crashes [15]. Communities located along the N3 were purposely selected for the study due to their high crash rates. Additionally, residents living along the N3 are often the first to arrive at injury scenes, making their involvement crucial for timely emergency response (Fig 1). Injury-prone areas along the N3 were mapped using Natural Earth data [16], which are in the public domain and freely available for use, distribution, and adaptation without restriction.

### Sample size, sampling and data collection

District health officials and community leaders in the Centre, Littoral and Southwest Regions of Cameroon assisted the research team to identify crash-prone areas across 11 health districts (Limbe, Tiko, Dibombari, Bonasama, Deido, Japoma, Edea, Pouma, Ngog mapubi Mbankomo

and Efoulan) spanning the N3 with high rates of RTIs. The Cochrane formula was used to estimate a sample size of 449, assuming a 50% expected proportion for willingness to provide prehospital care, as no previous study was available, to maximize the sample size with a 5% margin of error at a 95% confidence interval. Trained Cameroonian national research assistants approached every 4^th^ house in each larger communities and purposely selected those living close to the road-axis in smaller communities to participate in the survey. In each community, a household representative aged 21 years or older, the legal age of adulthood in Cameroon, was approached for survey consent. All adults permanently living in the designated community of the N3 across rural (quiet, open spaces with fewer people, buildings, and mainly farming) and urban (busy places with lots of buildings, roads, businesses, and people) settings for at least six months were eligible for inclusion. This non-probability sampling approach aimed to capture diverse perspectives on injury exposure in varying geographic contexts. Consenting participants completed a verbally administered closed-ended structured questionnaire gathering data on: sociodemographic data, injury and first-aid training experience, current knowledge on first-aid methods, attitudes and willingness regarding participation in provision of prehospital care for injury.

### Determination of first-aid knowledge and willingness to provide prehospital care

The questionnaire was developed by the study team and pre-tested on 20 community members living along National Road number 8 in Buea, South West Region of Cameroon. Questions on ﬁrst aid knowledge and willingness to provide prehospital care was adapted from a similar study conducted in Ghana [17]. Knowledge of first-aid techniques was assessed using 11 questions, with single-response items scored as 1 point each, while multiple-response items were proportionally weighted so that the total score for each question summed to 1 based on the number of correct answers. The level of knowledge on first-aid methods was categorized using Bloom’s criteria based on the percent of correct responses to questions: below 80% was classified as inadequate and ≥80% as adequate knowledge. This threshold aligns with educational standards in KAP assessments and has been applied in similar studies, including a 2025 Ugandan study on burn first-aid knowledge that used the same ≥80% cutoff for adequacy [18] Willingness to provide prehospital care as lay first responder was assessed based on a combination of three questions. Participants were asked to report their willingness to participate in prehospital care if (1) the victim was a stranger, (2) the participant was the only person who can help in the injury scene, and (3) other people being present at the injury scene. Responses ranged from “strongly disagree” (1 point) to “strongly agree” (5 points). We defined persons as being willing to provide prehospital care if they either agreed or strongly agreed to all three questions. The three-item willingness index showed acceptable internal consistency (Cronbach’s alpha = 0.87). Score distributions were reviewed to assess for ceiling effects, and while 26.5% of participants scored the maximum, the scale retained sufficient variability for meaningful analysis.

### Statistical analysis

Survey data were summarized using descriptive statistics including frequencies and percentages (%) for categorical variables, and medians and Interquartile Range (IQR) for continuous variables. Comparisons between groups were calculated using the chi-square test of independence for categorical variables. The enter method of multivariable logistic regression model was used to explore associations between demographics, injury exposure, and first-aid knowledge with the willingness to provide prehospital care. For all analyses the alpha level of statistical significance was set at p < 0.05 and the IBM-SPSS version 26.0 software was utilized for data analysis.

### Ethical considerations

Ethical approval for the study was obtained from the Faculty of Health Sciences Institutional Review Board of the University of Buea (reference number: 2024/2482-03/UB/SG/IRB/FHS) and the Centre Regional Ethics Committee for human Health Research (reference number: 0623/CRERSHC/2024). Administrative authorization was obtained from the Regional Delegation of the Ministries of Public Health for the Southwest, Littoral and Centre Regions in Cameroon. Prior to data collection, the purpose of the study was explained to participants and written informed consent obtained from each respondent.

## Results

### Socio-demographic characteristics of respondents

A total of 449 community members from 64 communities participated in the study across 11 health districts in 3 geopolitical regions (Fig 1). The median age of the participants was 33 years (interquartile range: 26–40), ranging from 21 to 70 years. The highest proportion of respondents were aged between 21 and 30 years. Most 268 (59.7%) of the participants were males, and 238 (53.0%) had attained a secondary level of education. The community members were mostly [268 (59.7%)] self-employed, with 235 (52.3%) reportedly having an estimated monthly income below 50,000 FCFA. Most, 267 (59.5%) of the respondents were living in rural areas along the N3. The socio-demographic characteristics of the study participants are presented on Table 1.

**Table 1:** Sociodemographic characteristics of residents along N3 highway in Cameroon.

Variable	Frequency (n = 449)	Percentage (%)
**Age group (years)**		
21-30	201	44.8
31-40	141	31.4
41-50	62	13.8
> 50	45	10.0
**Sex**		
Male	268	59.7
Female	181	40.3
**Level of Education**		
Primary	129	28.7
Secondary	238	53.0
University	69	15.4
None	13	2.9
**Main occupation**		
Self-employed	268	59.7
Private sector employee	117	26.1
Student	30	6.7
Unemployed	19	4.2
Public sector employee	11	2.4
Housewife	4	0.9
**Estimated monthly income**		
< 50,000	235	52.3
50,000 - 100,000	94	20.9
101,000 −150,000	76	16.9
150,000 +	44	9.8
**Area of residence**		
Rural	267	59.5
Urban	182	40.5

### Willingness to provide prehospital care to victims of road traffic injuries

**Fig 2:**
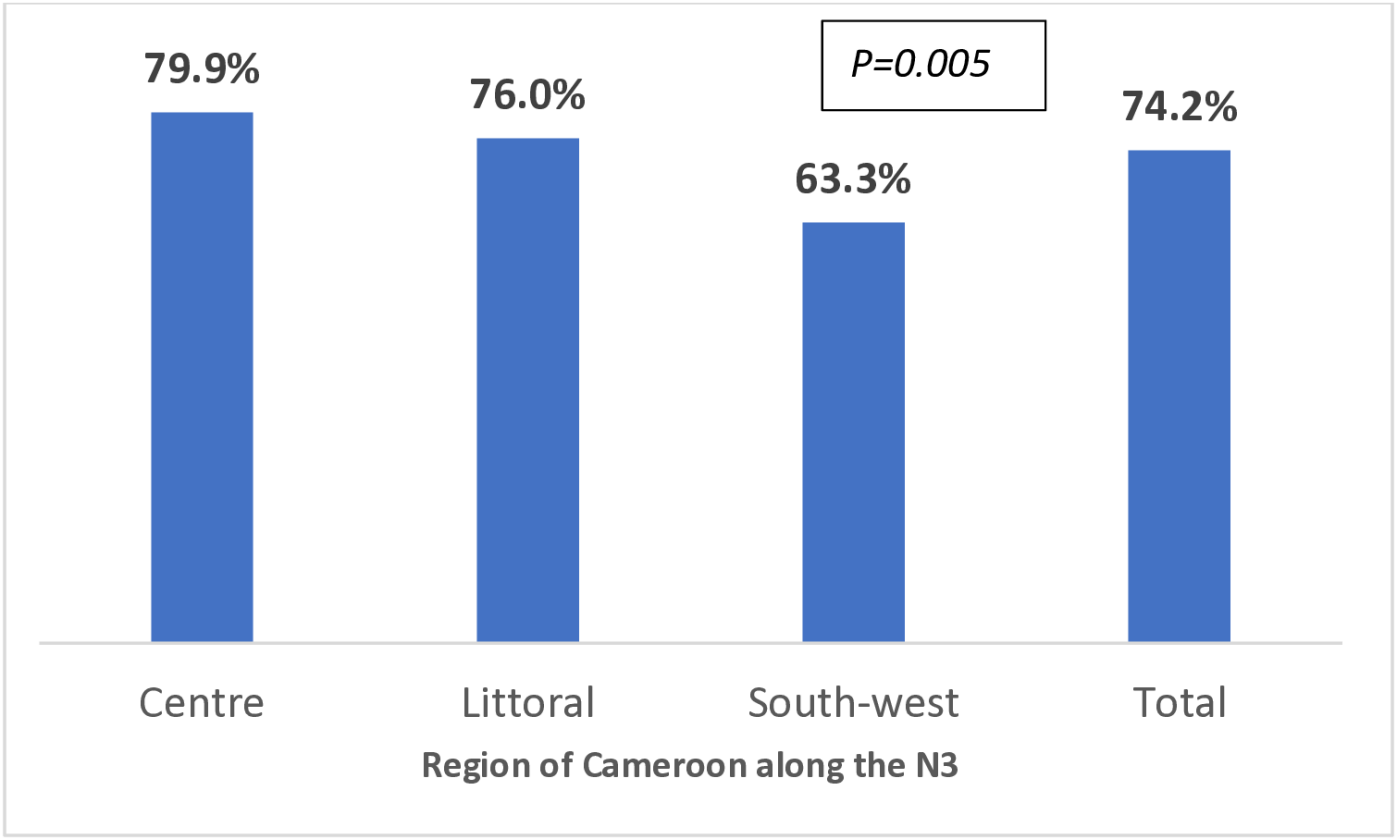
Willingness to provide prehospital care to victims of road traffic injuries among local residents living along N3 highway in Cameroon.

The majority, 74.2% of the community members were willing to provide prehospital care for road traffic injuries in a LFR program. Local residents along the N3 within the Centre region (79.9%) were significantly more willing to participate in the provision of prehospital care compared to their counterpart within the South-west region (63.3%) of Cameroon (p = 0.005) (Fig 2).

### Community preferences for participation in a lay first responder program

Most respondents (87.1%, n = 391) reported willingness to report RTIs, mainly as pictures of the crash (55%) and phone calls (53%). Data credit was the most (73%) preferred resource needed to report injuries. Most respondents (92%) expressed the need to be trained on basic first aid methods to participate a LFR program (Table 2).

**Table 2:** Community preferences for participating in a lay first responder program for road traffic injuries along N3 highway in Cameroon (n = 449).

Variable	Category	Frequency (%)
**Preferred means of reporting injury**		
	Photograph of crash	248 (55)
	Phone call	239 (53)
	Text message	143 (32)
	Voice message	130 (29)
**Preferred resources needed to report injury**		
	Communication credit	325 (72)
	Mobile phone	275 (61)
	Internet bundle	259 (58)
	Reporting tool	103 (23)
**Preferred support to participate in a LFR program**		
	Training on basic first aid methods	414 (92)
	Provision with needed resources	397 (88)
	Offering of incentives	192 (43)

### Knowledge of emergency helplines and experiences of community members living along N3 highway in Cameroon

In the course of the last 12 months before to the study, 202 (45.0%) of the surveyed community members reported having witnessed a road traffic crash. About a third [136 (30.3%) of the community members had encountered an injury situation that required calling emergency response services, largely due to RTI (Table 3).

**Table 3:** Experience of participants in reporting road traffic injuries along N3 highway in Cameroon (n = 449).

Variable	Category	Frequency (%)
**Aware of emergency helpline numbers**	No	280 (62.4)
	Yes	169 (37.6)
**Encountered injury requiring need to call emergency responders**	No	313 (69.7)
	Yes	136 (30.3)
**Injury type**	Road traffic crash	95 (69.9)
	Fire emergency	36 (26.5)
	Domestic accident	24 (17.6)
	Fall from high	3 (2.2)
	Industrial accident	6 (4.4)

**Community members’ knowledge of first aid for injured victims along the N3 highway** Most (86%, n = 386) of the N3 residents had heard about first aid, but only 5% reported to have had any education on ﬁrst aid in the past 12 months. Based on the 11 categories of questions about ﬁrst aid captured in the study, only 167 (37.2%) of community members had adequate knowledge (≥80% of the total knowledge scores) of ﬁrst aid. There was no association between prior education on ﬁrst aid methods and adequate ﬁrst aid knowledge; 36.6% for residents without prior ﬁrst aid education versus 47.8% for participants with prior ﬁrst aid education (p = 0.279). Most respondents (92.7%, n = 416) recognized injuries accompanied by bleeding to require first aid. The most widely recognized first aid measures among respondents were pressing firmly on a wounded site with a clean bandage to stop bleeding (93.1%, n = 418), prioritizing the control of bleeding in first aid (92.4%, n = 415), and applying direct pressure on a wound to stop external bleeding (90.4%, n = 406). A small proportion of respondents recognized the importance of checking for a pulse (47.2%) and avoiding shaking an injured person (45.4%) as essential first aid measures (S1 Table).

### Community attitudes toward providing first aid to RTI victims along the N3 highway

Most community members (76%, n = 340) demonstrated positive attitudes towards participation in a LFR program, notably of the opinion that providing scene care increase chances of survival (96%), learning about first-aid (99%), willing people trained to provide prehospital care (94%) and laypersons have the responsibility to provide first aid (81%). However, only 55 (12%) of the respondents had heard about the good Samaritan law.

### Factors associated with willingness to provide prehospital care to RTI victims along N3 highway

Variables that showed a significant association (p < 0.05) with willingness to provide prehospital care in the bivariate analysis were included in a multiple logistic regression model. The logistic regression model was statistically significant (χ² = 51.37, p < 0.001), explaining 15.9% of the variance in willingness (Nagelkerke R² = 0.159) with good model fit (Hosmer-Lemeshow χ² = 6.10, p = 0.636). Variables that were found to be associated with willingness to provide prehospital care on bivariate regression included having adequate first aid knowledge, being aged 31–40 or 50–60 years, male gender, possessing a primary or secondary level of education, residing in a rural area, having witnessed a road traffic crash in the past year, and being aware of Good Samaritan laws. In multivariate logistic regression only having sufficient first aid knowledge, being aged between 31 and 40 years, possessing primary or secondary education, witnessing a road traffic crash in the past year, and expressing the belief that community members should be able to call emergency medical services to report emergencies remained statistically associated with willingness to provide prehospital care to victims of RTIs (Table 4).

**Table 4:** Factors associated with willingness of community members living adjacent N3 to provide prehospital care for RTIs.

Variable	aOR	95% CI	p-value
**Knowledge on First Aid**			
Inadequate [Ref.]			
Adequate	1.69	1.01 - 2.81	0.046
**Age group (years)**			
21-30 [Ref.]			
31-40	1.82	1.06 - 3.14	0.031
41-50	1.64	0.79 - 3.41	0.183
>50	2.59	0.92 - 7.30	0.072
**Sex of respondent**			
Female [Ref.]			
Male	1.55	0.98 - 2.45	0.061
**Level of Education**			
Primary	4.20	1.19 - 14.81	0.026
Secondary	4.70	1.34 - 16.53	0.016
Tertiary	3.41	0.89 - 13.00	0.073
None [Ref.]			
**Residence type**			
Urban [Ref.]			
Rural	1.58	0.99 - 2.55	0.057
**Witnessed RTI during the past 12 months**			
No [Ref.]			
Yes	1.68	1.055 - 2.68	0.028
**Feel it is need to call EMS for an emergency**			
No [Ref.]			
Yes	3.11	1.28 - 7.54	0.012
**Heard of “The good Samaritan Law”**			
No [Ref.]			
Yes	1.88	0.78 - 4.53	0.159

[Ref.] = reference category.

## Discussion

As part of a broader initiative to establish a scalable lay first responder prehospital care program in Cameroon, we conducted a cross-sectional study to evaluate the willingness of community members to report and participate in injury response and to identify demographic and experiential factors associated with willingness to participate. We found that respondents were overwhelmingly willing to report injury and participate in injury response, suggesting that LFR training would likely be highly acceptable within this community. Furthermore. we found that increased knowledge of first aid was associated with willingness to participate in injury response. These data support for the hypothesis that injury-exposed communities in Cameroon may be a high-impact target cohort for LFR training to increase prehospital care and improve survival among Cameroonian trauma victims.

In other resource-limited sub-Saharan nations, LFR programs training commercial drivers and police have demonstrated promising educational efficacy and retention in first aid knowledge and responder satisfaction [19–21]. In the Cameroonian context, commercial drivers provide most of the transport for patients injured in road traffic incidents and were initially targeted as an ideal LFR cohort. However, acceptability studies for this cohort demonstrated a mixed picture, where the success of the program would likely be predicated on driver compensation and improvement in healthcare worker-driver relations. Our study provides the first data from Cameroon evaluating the contextual-fit of a non-driver cohort for LFR training and suggests that training communities along perilous throughways like the N3 may be a high-impact intervention to increase prehospital care. Although variable in recruitment, community-based first-aid interventions have been reported in both low and high-income settings, including Congo [22], Zambia [23], South Africa [24], and the United States [25]. These data highlight that potential first aid training program must be tailored to local injury patterns and cultural contexts to increase engagement.

Factors associated with willingness to intervene in road traffic incidents re-demonstrates the potential impact of training interested community members. A lack of awareness of emergency systems was apparent, as only a few respondents knew about emergency helplines, while most preferred seeking help from neighbors rather than professional responders. This highlights broader systemic shortcomings, where informal networks often take the place of formal emergency services due to a lack of adequate infrastructure. This may be especially true in rural communities, where respondents were more willing to offer first aid themselves. Community members may be incentivized to participate knowing that they may serve as the temporizing measure to provide stabilization for injured patients prior to transport to more formalized medical settings. Beyond the initial sample size estimation using Cochrane formula, a post hoc evaluation of the model’s performance supports its adequacy for exploratory analysis. The model demonstrated sufficient explanatory strength to identify meaningful associations, particularly for education, age, and first-aid knowledge. This suggests these variables are relevant factors in understanding willingness to serve as LFRs in community settings. While not a substitute for formal power calculations, the model’s fit and consistency reinforce the credibility of its findings and provide a strong foundation for future confirmatory research.

This study has several notable limitations. Due to the cross-sectional study design, we cannot infer causality between any demographic or experiential factors and willingness to participate in injury response. Longitudinal studies could provide deeper insights into how willingness evolves over time. Secondly, self-reported data may be influenced by social desirability bias, potentially overestimating willingness to participate. Thirdly, the study recruited self-identified representatives of household which largely consisted of males owing to cultural practices where the male figures speak on the behalf of their families. Given that health-related behaviors can vary significantly by gender, the male-dominant sample may not accurately reflect the perspectives of women in the community. Willingness to participate among household heads may not perfectly reflect the perspectives of other potential lay-first responders in N3 adjacent communities. A moderate ceiling effect was observed in the willingness index, with over 26% of participants scoring the maximum. This suggests limited sensitivity in detecting variability among highly willing individuals and may have led to an overestimation of readiness to intervene. Social desirability bias may have led participants to overstate their willingness to participate, aligning with perceived community expectations. This could overestimate actual intentions. Future studies should consider too refinement, triangulating with qualitative methods and using self-administered tools to reduce such bias. While purposeful selection may introduce non-random sampling bias, it was chosen to ensure the inclusion of participants from both high-risk rural and urban contexts along the N3 corridor. This approach prioritizes contextual relevance over generalizability, which is appropriate for exploratory, community-based studies aimed at identifying locally relevant intervention opportunities. Inclusion of qualitative data could strengthen future analyses by providing more nuanced understanding of the barriers and potential facilitators of LFR in this community. Furthermore, the study focused on communities along a single national road (N3), limiting the generalizability of findings to other regions of Cameroon with differing socio-economic or socio-cultural contexts. Expanding the geographic scope would enhance the representativeness of future research.

These findings provide valuable direction for the development of a LFR program, emphasizing community-based first-aid training and practical emergency skills to drive long-term behavioral change. Given the absence of a unified emergency response number, the program should prioritize clear guidance on transport decisions to enable quick access to definitive trauma care. Integrating digital tools, such as mobile apps with voice and photo-sharing capabilities, can enhance reporting and first-aid support. Additionally, mobilizing rural communities where willingness is higher in addition with providing incentives like free training and communication credits, can boost participation. Finally, incorporating real-time clinical data and longitudinal acceptability studies will be crucial for assessing effectiveness and impact, addressing a gap that many previous studies have overlooked due to data limitations.

## Conclusion

The willingness of community members along N3 highway to provide prehospital care for victims of RTIs is shaped by socio-demographic, knowledge, and attitudinal factors. Addressing barriers through targeted training and systemic improvements can transform positive attitudes into actionable behaviors. While these findings align with global trends, regional nuances underscore the importance of localized solutions. Future research should adopt longitudinal designs and broader geographic scopes to build on this study’s insights, guiding comprehensive strategies to enhance establishment of lay first responder systems to reduce RTI-related morbidity and mortality in Cameroon.

## Supporting information

S1 Table Knowledge of respondents in communities along N3 highway in Cameroon regarding provision of first aid. (DOCX)
