# Supplementary material for: Community willingness to participate in prehospital injury care: A cross-sectional survey of injury-prone areas along the national 3 highway in Cameroon
Source: PLoS One. 2025 Sep 11;20(9):e0332179. doi: 10.1371/journal.pone.0332179 (PMC12425217; doi:10.1371/journal.pone.0332179)
Supplement: S1 Table — (DOCX) [file pone.0332179.s001.docx]

**Supplementary material**

**S1 Table: Knowledge of respondents in communities along N3 highway in Cameroon regarding provision of first aid**

| **Correct first-aid knowledge** | **Frequency (%)** |
| --- | --- |
| Injuries accompanied by bleeding need first aid | 416 (92.7) |
| Fracture sustained from injuries need first aid | 338 (75.3) |
| Injuries sustained from human or animal bite need first aid | 374 (83.3) |
| Injuries from burns need first aid | 362 (80.6) |
| Neck, back and spine injuries need first aid | 227 (50.6) |
| Injuries resulting to breathing difficulties need first aid | 314 (69.9) |
| Pressing firmly on a wounded site with a clean bandage is a measure to stop bleeding | 418 (93.1) |
| Placing an unconscious but breathing patient in the recovery position is a first aid protocol | 395 (88.0) |
| Avoiding head and neck movement and keeping the body straight for persons with neck and back injury is a first-aid method | 335 (74.6) |
| Pouring water on an unconscious injured person will not help them recover faster | 182 (40.5) |
| Call for an ambulance before removing any hazards when road traffic crash occurs | 30 (6.7) |
| Injured persons should be transported to the hospital after road traffic crashes | 120 (26.7) |
| Check for the pulse of an unresponsive patient at the neck | 212 (47.2) |
| Do not check for responsiveness from an injured person by shaking the victim | 204 (45.4) |
| Place unresponsive but breathing victims in the recovery position | 377 (84.0) |
| Apply direct pressure on the wound to stop external bleeding | 406 (90.4) |
| Chest compression is a first‐aid priorities | 362 (80.6) |
| Maintaining breathing is a top priority in first aid. | 366 (81.5) |
| Stopping bleeding is a first‐aid priorities | 415 (92.4) |
